# Heavy-load exercise in older adults activates vasculogenesis and has a stronger impact on muscle gene expression than in young adults

**DOI:** 10.1186/s11556-022-00304-1

**Published:** 2022-10-01

**Authors:** Kaare M. Gautvik, Ole K. Olstad, Ulrika Raue, Vigdis T. Gautvik, Karl J. Kvernevik, Tor P. Utheim, Solveig Ravnum, Camilla Kirkegaard, Håvard Wiig, Garan Jones, Luke C. Pilling, Scott Trappe, Truls Raastad, Sjur Reppe

**Affiliations:** 1 Unger-Vetlesen Institute, Lovisenberg Diaconal Hospital, Oslo, Norway; 2 Department of Medical Biochemistry, Oslo University Hospital, Oslo, Norway; 3 Human Performance Lab, Ball State University, Muncie, IN USA; 4 Department of Plastic and Reconstructive Surgery, Oslo University Hospital, Oslo, Norway; 5 Department of Ophthalmology, Stavanger University Hospital, Stavanger, Norway; 6 Department of Ophthalmology, Sørlandet Hospital Arendal Surgical Unit, Arendal, Norway; 7 Department of Physical Performance, Norwegian School of Sports Sciences, Oslo, Norway; 8 College of Medicine and Health, University of Exeter, Exeter, UK

**Keywords:** Muscle, Transcriptome, Exercise, Older adults, Muscle biopsies

## Abstract

**Background:**

A striking effect of old age is the involuntary loss of muscle mass and strength leading to sarcopenia and reduced physiological functions. However, effects of heavy-load exercise in older adults on diseases and functions as predicted by changes in muscle gene expression have been inadequately studied.

**Methods:**

Thigh muscle global transcriptional activity (transcriptome) was analyzed in cohorts of older and younger adults before and after 12–13 weeks heavy-load strength exercise using Affymetrix microarrays. Three age groups, similarly trained, were compared: younger adults (age 24 ± 4 years), older adults of average age 70 years (Oslo cohort) and above 80 years (old BSU cohort). To increase statistical strength, one of the older cohorts was used for validation. Ingenuity Pathway analysis (IPA) was used to identify predicted biological effects of a gene set that changed expression after exercise, and Principal Component Analysis (PCA) was used to visualize differences in muscle gene expressen between cohorts and individual participants as well as overall changes upon exercise.

**Results:**

Younger adults, showed few transcriptome changes, but a marked, significant impact was observed in persons of average age 70 years and even more so in persons above 80 years. The 249 transcripts positively or negatively altered in both cohorts of older adults (q-value < 0.1) were submitted to gene set enrichment analysis using IPA. The transcripts predicted increase in several aspects of “vascularization and muscle contractions”, whereas functions associated with negative health effects were reduced, e.g., “Glucose metabolism disorder” and “Disorder of blood pressure”. Several genes that changed expression after intervention were confirmed at the genome level by containing single nucleotide variants associated with handgrip strength and muscle expression levels, e.g., *CYP4B1* (*p* = 9.2E-20), *NOTCH4* (*p* = 9.7E-8), and *FZD4* (*p* = 5.3E-7). PCA of the 249 genes indicated a differential pattern of muscle gene expression in young and elderly. However, after exercise the expression patterns in both young and old BSU cohorts were changed in the same direction for the vast majority of participants.

**Conclusions:**

The positive impact of heavy-load strength training on the transcriptome increased markedly with age. The identified molecular changes translate to improved vascularization and muscular strength, suggesting highly beneficial health effects for older adults.

**Supplementary Information:**

The online version contains supplementary material available at 10.1186/s11556-022-00304-1.

## Background

Increasing fragility of the musculoskeletal system is a serious threat to healthy ageing. Muscular weakness, or sarcopenia, due to inactivity and illness may impose a heavy burden on life quality of those affected [1]. The annual cost of hospitalizations in US adults with sarcopenia amounted to USD 40.4 billion in 2019 with an average per person cost of USD 260 [2]. In the Nordic countries, women live longer than men, with a mean anticipated lifespan of ~ 84 years, and the health costs associated with weakened muscular power facilitating imbalance, falls, and fractures due to longevity are very high and rising [3].

Resistance training has been previously shown to improve muscle mass and strength as well as many other health and quality of life related parameters in various groups of older individuals [4–6]. However, knowledge of muscle gene regulation in postmenopausal (PM) women during prolonged heavy-load exercise and how transcriptome changes may translate to mechanical strength, is scarce. Most studies are of shorter durations and/or involve younger people, often comprising patients with metabolic syndrome or hormonal diseases focusing on a select number of genes, proteins, and/or metabolites [7]. Increased knowledge of basic muscle biology and genetics addressing the lack of understanding of the molecular machinery involved, and how it translates to loss of muscle mass and mechanical strength, is a prerequisite for preventive and therapeutic interventions. Moreover, we hypothesized the information obtained in the current study would be able to explain, at least partly, the phenomenon of sarcopenia.

To date, the most far-reaching investigation of gene regulation at the transcriptional level in muscle tissue comparing exercise and inactivity, is the MetaMex meta-study using 66 published datasets [7, 8]. The MetaMex study and online resource https://www.metamex.eu/ are excellent for looking up genes affected by various forms of training. However, only 13 datasets mimic the current long duration supervised resistance-exercise training (RET) study with muscle transcriptome analysis before and after the training period. It should also be noted that the MetaMex datasets involved smaller, heterogeneous cohorts of young and old, women and men in studies of different design and sizes. In all, only 213 persons were included in the 13 studies/datasets [7]. One of the most comprehensive of the 13 RET studies, performed by Raue et al. [9] (BSU cohorts) focused on effects of exercise on the various fiber types. Fiber type specific microarray analysis, although measured as acute response, showed that the vast majority of gene changes occurred in fast-twitch (MHC IIa) muscle fibers and that the young persons had a more profound increase in fast-twitch (MHC IIa) muscle fiber hypertrophy after 12 week RET. However, they also showed that the number of genes changed in total/mixed vastus lateralis muscle biopsies after 12 week RET was much higher in the elderly as compared to young. Thus, warranting further studies in larger cohorts.

In the current study we combined thigh muscle transcriptome datasets provided by Raue et al. [9] with similar datasets from the Oslo cohort (briefly described below and by Olstad et al. [10]. We were thus able to use larger datasets with stronger statistical power, to answer novel research questions such as what is the functional impact of heavy-load exercise in young and elderly, and also evaluate functional differences between the age groups. Using novel public data on gene variants associated with muscle expression levels (expressed quantitative loci) (eQTL) and handgrip strength we could also answer questions such as which are the causal genes responsible for some of the observed effects.

The present study is a follow-up of our recent paper by Olstad et al. [10] in which the main findings were that osteoporotic and healthy women have distinct muscle transcriptomes, before and after RET, and that OP women had a higher proportion of type I fibers. We first addressed the genetic and molecular mechanisms in the muscle tissue of 35 postmenopausal women using a global approach covering all acknowledged genes (the transcriptome) to delineate the most important changes following prolonged RET. The present study is the largest uniform RET study of older women enabling the study of transcriptional changes specific for this population at highest risk for sarcopenia. Thigh muscle biopsies were examined before and after 12–13 weeks of heavy-load intervention training in women without metabolic or cardiovascular diseases. The data were exploited to identify the genes that were statistically most affected to understand the exact nature of the molecular networks involved. The results were validated in the BSU cohort of older adults. Also, in order to define the specific transcriptional deficiency in elderly associated with sarcopenia, we used comparable data from young adults obtained before and after exercise since they do not experience a similar muscle wasting condition. Furthermore, DNA nucleotide variants or Single Nucleotide Polymorphisms (SNPs) were looked up in accessible databases for associations with levels of specific transcripts in muscle (expressed quantitative loci (eQTL)) known to affect the most training-sensitive muscle genes. Finally, we tested whether these SNPs were associated with low grip strength/weakness, a characteristic sarcopenic phenotype in females using published data [11].

## Methods

### Participants: Recruitment, analyses, and ethics

**Fig. 1:**
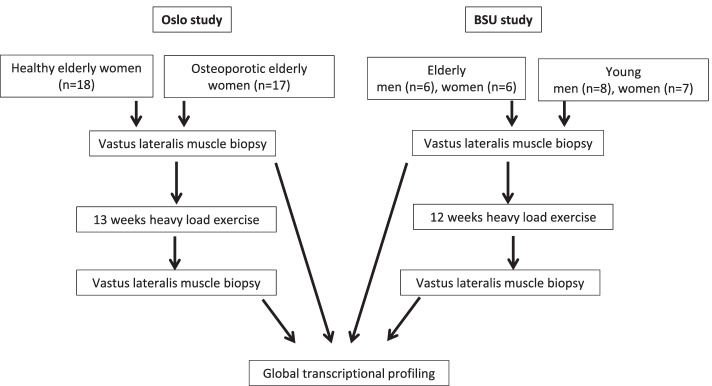
Outline of study

Cohorts from two studies were used in this paper as outlined in Fig. 1. The Oslo cohorts of PM women were recruited consecutively through an outpatient medical clinic. The women gave their verbal and written consent as described previously [12]. These cohorts represented healthy women (*n* = 18) or women with established primary osteoporosis (*n* = 17), (age, 55–80 years) without cardiovascular, endocrine, or neurological diseases. The osteoporotic women received anti-resorption medication (bisphosphonates), which was withdrawn 3 months prior to starting this study. The number of previous smokers and the level of physical activity were similar between the groups, as were other lifestyle factors and nutrition as previously described [12]. The serum and urine biomarkers of all participants were normal. Clinical evaluation: All participants underwent a clinical examination and completed detailed interview questionnaires on present and previous diseases, nutrition, and lifestyle factors (smoking, alcohol, physical activity). All participants received daily supplements of vitamin D3 (1000 IU) and calcium (1000 mg). The Ball State University (BSU) cohorts have been extensively described previously [9] but are summarized below and in Table 1. BSU participants were excluded based on similar criteria as the Oslo cohort: any acute or chronic illness, cardiac, pulmonary, liver, or kidney abnormalities, uncontrolled hypertension, insulin- or non-insulin-dependent diabetes, abnormal blood, or urine chemistries, arthritis, a history of neuromuscular problems, or if they smoked tobacco. From the BSU cohorts comprising 12 old donors (6 women, 6 men, average age 84 ± 3 years) or 15 young donors (7 women, 8 men, average age 24 ± 4 years), we used transcriptome data based on thigh muscle biopsies taken before the first and before the last exercise in their 12-week training period. The data are available from GEO: https://www.ncbi.nlm.nih.gov/geo/query/acc.cgi?acc=GSE28422

**Table 1:** Demographic characteristics of muscle biopsy donors

	**Group**			
	Oslo (*n* = 35)	BSU old (*n* = 12)	BSU young (*n* = 15)	Donors of iliac muscle (*n* = 24)
Age, years	70.1 (6.3)	84 (3)	24 (4)	67.4 (11,0)
Body mass, kg	68.1 (11.0)	70 (9)	72 (13)	68.5 (12.4)
BMI	24.1 (3.9)	26 (3)	25 (5)	25.0 (3.6)
Lean mass, kg	40.8 (4.3)			40.5 (4.9)
Fat mass, kg	23.9 (3.7)			23.7 (7.7)
Maximum squat load, kg	35.1			

The Oslo group and donors of iliac muscle included women only, while The BSU groups contained women and men (see Methods). Numbers indicate mean with standard deviation (SD)

*BMI* Body mass index

### Training protocols

Oslo Cohorts: The total duration of the intervention was 13 weeks for healthy and 15 weeks for the patients with primary osteoporosis because the first two weeks were used to familiarize them with the training protocol starting with lighter training loads. The training loads were gradually increased to ensure that the 13 weeks of training were conducted with optimal loading to improve muscle strength and muscle mass [12]. The training period was performed as traditional heavy-load strength training: three times per week with 1–3 sets involving all major muscle groups as detailed previously [12]. Briefly, the training protocol consisted of three exercises for the leg muscles (squat, leg press, and standing toe rise), and three exercises for the upper body muscles (chest press, seated rowing, and shoulder press). In addition, the participants performed self-selected exercises for the abdominal and lower back muscles at the end of each session. The strength-training regimen was a mix of linear periodization and daily undulating periodization. The participants started with 8–12 repetition maximum (RM) sets, and ended the 13-week protocol with 4–8 RM sets. In two sessions per week, the sets were run until failure (RM-sets); in the third session, performed between the two maximal sessions, sets were run with a load corresponding to 80–90% of the actual RM load. The total duration of training was about 60 min per session, and the participants exercised in groups of three with a personal instructor present.

The BSU cohorts underwent a 12-week training regimen with progressive resistance training (PRT). The groups performed a smaller, leg focused selection of exercises, but included 36 training sessions (3 days/week) with three sets of 10 bilateral knee extensions at 70–75% of their 1 RM [9]. Thus, training of the vastus lateralis muscle group was similar in the Oslo and BSU studies, although the load used in the BSU cohort was somewhat lower.

Questions may be asked if the transcription repertoire (the transcriptome) in the basal resting state is the same in mechanically loaded skeletal muscle (e.g., thigh) as in unloaded muscle (e.g., iliac/pelvic). If not, their responses to training could have been different, and the results would have been needed a further explanation. Thus, we compared if these muscles, serving vastly different functions (dynamic versus static), had the same transcriptional profile in the basal, rested state selecting muscle biopsies from pelvis of well characterized female donors (12 healthy and 12 with osteoporosis) with similar age and BMI and not part of a training study [13].

### Muscle biopsy collection and RNA purification

Biopsy collection and RNA purification from the Oslo and BSU cohorts used similar methods and technology, as previously described [9, 10]. In brief, thigh muscle biopsies were obtained under local anesthesia (xylocaine adrenalin, 10 mg/ml + 5 μg/ml; AstraZeneca, London, UK) from the mid portion of the vastus lateralis before and after the training period using a modified Bergström technique. The muscle samples were obtained at least 2 days after any training or testing, and the second biopsy was obtained approximately 3 cm distal to the previous site. The biopsies were taken from fasting participants of both studies in the morning (07–09 AM) to allow for similar levels of physical activity and dietary intake. A sample (10–20 mg) to be used for RNA extraction was immediately frozen in liquid nitrogen (applied to the cohort of osteoporotic women; muscle biopsies from all other participants were stored in RNAlater (Merck, Darmstadt, Germany) for 1 day at 4 °C before freezing. The samples were then stored at -80 °C until RNA purification using a RNeasy Mini Kit (Qiagen, Oslo, Norway) according to the manufacturer’s instructions.

### Microarray and data analysis

The same type of microarray analysis was performed on the BSU and Oslo cohorts employing Affymetrix HG-U133 Plus 2.0 or Affymetrix HuGene-1_0-st-v1 arrays (Thermo Fisher Scientific, Waltham, MA, USA). Robust microarray analysis (RMA) yielding normalized log2 transformed signal intensities was applied for normalization for both array types. (http://bip.weizmann.ac.il/toolbox/overview/Partek_Users_Guide.pdf). Gene transcripts with maximal signal values of < 5 (log2 values) across all arrays were removed to filter for low- and non-expressed genes. Differentially expressed transcripts before vs. after training were identified using two-way analysis of variance (ANOVA) as implemented in Partek Genomics Suite (Partek, St. Louis, MO, USA).

Further bioinformatics analysis on thigh muscle biopsies was conducted on the significant genes to identify functional implications with Ingenuity Pathway Analysis (Ingenuity Systems, Redwood City, CA, USA). The microarray data generated from the Oslo and BSU cohorts have previously been validated using real-time qRT-PCR on selected genes [9, 11].

### Comparison of basal iliac and thigh muscle transcriptomes

In a previous study [13] we collected postmenopausal trans-iliac bone biopsies using a Bordier trephine [14]. Iliac muscle attached to the pelvic side of these bone biopsies were separated and snap frozen in liquid nitrogen (*n* = 24). Subsequent RNA isolation was performed as described for thigh muscle biopsies. However, Affymetrix HG-U133 Plus 2.0 arrays were used for transcriptome profiling, while HuGene-1_0-st-v1 arrays were used for transcriptome profiling of thigh muscle RNA. Use of different array types prevented us from normalizing the two datasets together. However, we conducted a Pearson correlation analysis of the Log_2_ transformed signal values using the 17 652 transcripts present in both datasets (93.5% of all thigh muscle transcripts). To visualize the similarity the average signal values for the 17 652 transcripts were plotted against each other (Fig. S1). As a further measure of similarity/difference between the iliac and thigh muscle transcriptomes a paired T-test was performed on the datasets.

### Functional enrichment analysis

Functional enrichment analysis identifies trends in large scale biological datasets and determines whether some functions are enriched in our set of differentially expressed genes. We used Ingenuity Pathway Analysis (IPA) (Qiagen, Beverly, MA, USA) for this task. Fisher’s exact test was used by IPA to identify enriched gene sets with all genes on the Human Gene 1.0 ST Array as the background gene list. Further information is found in the Table 2 legend.

**Table 2:**
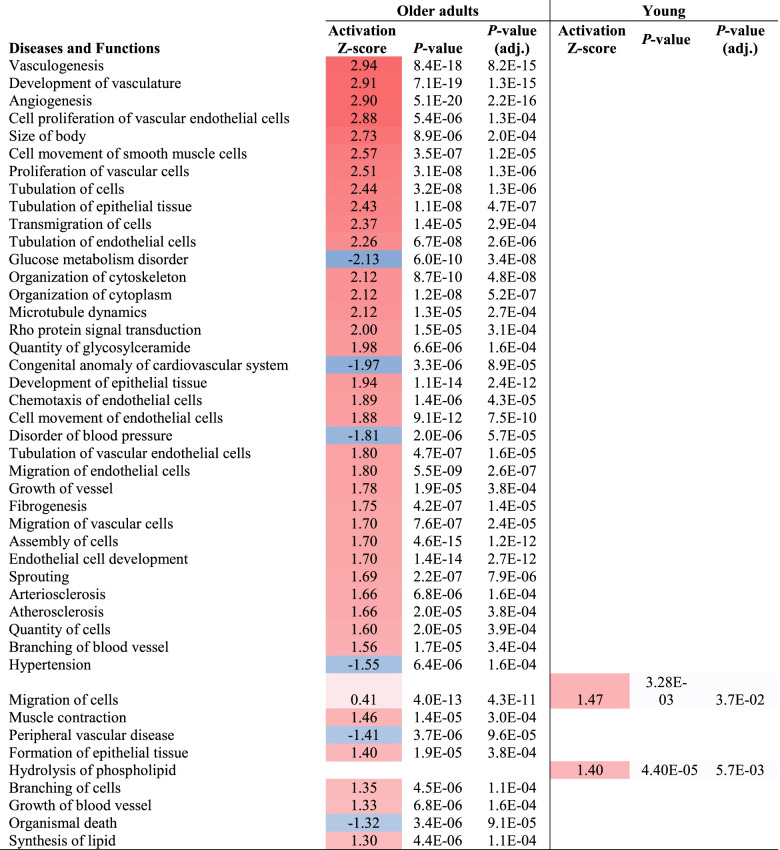
Over-represented diseases and functions

The table data were extracted from the “Comparison Analysis” function in IPA. The table illustrates common genes from the Oslo and BSU study changing expression (end vs start of intervention period) at q-value < 0.1 in Older adults, and the genes shifting expression in the cohort of young from the BSU study (q-value < 0.2). In this analysis, we excluded Diseases and Functions with activation Z-score <|1.3|, all with adj. *p*-value > 0.05, and all diseases/functions related to cancer

### Principal component analysis (PCA)

PCA is a frequently used dimensionality-reduction method with the aim to reduce the dimensionality of large data sets by transforming extra sized sets of variables into smaller ones that still contain most of the information. We used the PCA generator as implemented in the software Partek Genomics Suite (Partek, St. Louis, MO, USA).

### Volcano plot

The volcano plot was generated using the volcano plot generation module in Partek Genomics Suite 6.6 (Partek).

### Analyses of association between transcripts responsive to heavy-load exercise training with muscle eQTLs and loci for handgrip strength in women

We identified SNPs associated with skeletal muscle expression for the genes listed in Table S1 using eQTL databases from the Genotype-Tissue Expression (GTEx) portal (https://gtexportal.org). Subsequently, significant SNPs were tested for association with low handgrip strength in females as summarized in a meta-analysis [11] adopting a nominal *p*-value < 0.01.

## Results

### Demographic characterization

Table 1 shows the demographic characteristics of all participants. The Oslo participants (*n* = 35) showed physical variables within the normal ranges for body mass, BMI, and lean and fat mass. Seventeen women had osteoporosis, and it is important to mention that they attained the same final maximal squat load as the others, i.e., 35.1 kg, after training. We combined the patients and healthy controls, testing first if their basal transcriptomes were inherently different using statistical comparisons.

The Oslo group and donors of iliac muscle were similar with regard to age, BMI, lean mass, fat mass, or mean body mass (Table 1), as well as serum laboratory analyses (not shown).

### Physical performance and muscle physiology variables after heavy-load exercise

A previous study [12] with the same female cohorts, showed that heavy-load strength training was feasible and equally effective for improving muscle mass and strength in osteoporotic and healthy with respect to increases in strength and muscle hypertrophy (mass), histology changes, muscle protein markers and type I and II fiber types and improvements of balance. Since the two groups achieved similar test results regarding physical performance and balance, physiological muscle effects, and stress protein responses to heavy-weight training, they were combined when studying the impact of RET on their transcriptomes.

### Training induced transcriptome changes in the Oslo and BSU cohorts of older adults

**Fig. 2:**
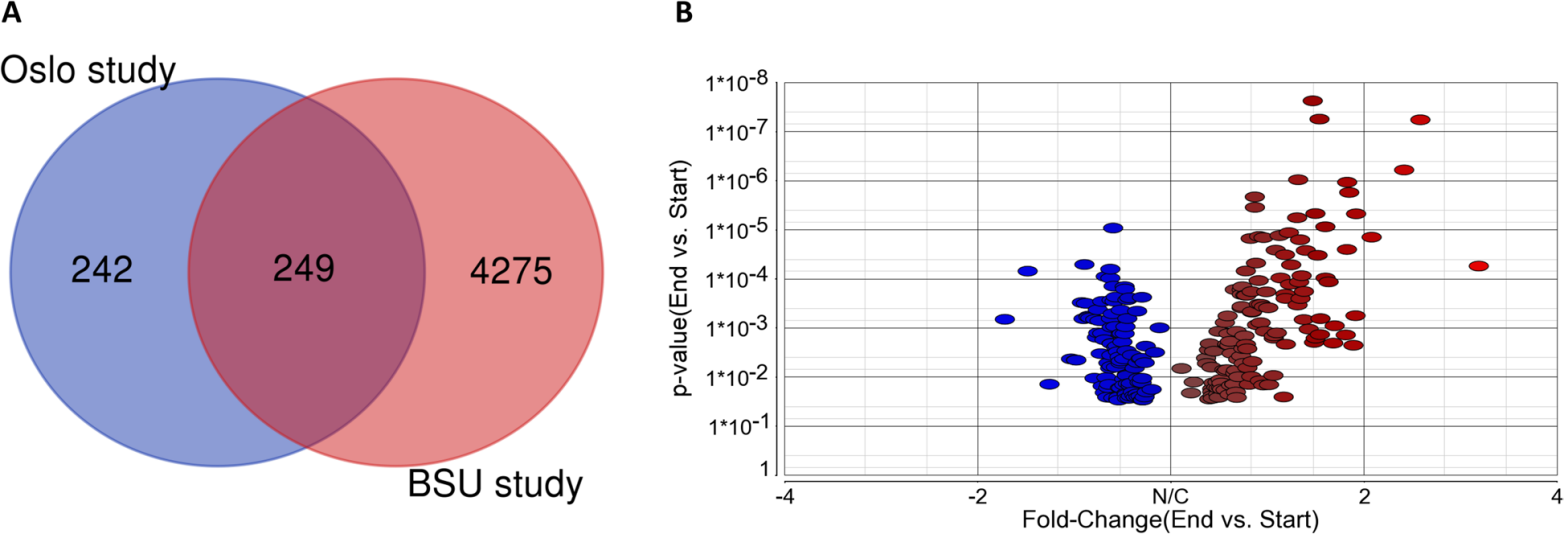
Training sensitive genes in the Oslo cohort are verified in the BSU cohort. **A** Venn diagram illustrating the fraction of common genes changing expression at Q-value < 0.1 after the training period in the Oslo and BSU cohorts, respectively. **B** Volcano plot showing the common training sensitive genes (*n* = 249) at q-value < 0.1 when comparing the end vs. start of intervention using linear regression analyses. The x-axis shows FC with positive (red) and negative (blue) values, respectively, when comparing individual transcripts, and the y-axis shows *p*-values

In the Oslo study, 491 annotated genes changed expression after the training period at Q-value < 0.1, while the corresponding number in the older BSU cohort was 4524. More than 50% of the more comprehensive Oslo study was replicated in the older BSU cohort (Fig. 2A). Figure 2B displays a volcano plot of 249 genes common for the two studies. Only four of the 249 common genes were changed in opposite directions between the two cohorts.

### Transcriptional changes in old vs young

Whereas 249 common genes were changed after the exercise period when comparing the older adults in Oslo vs older adults in BSU at q value < 0.1, only one gene (*PABPC4*) changed at q-value < 0.1 in the BSU cohort of young (78 genes were changed at q-value < 0.2).

**Fig. 3:**
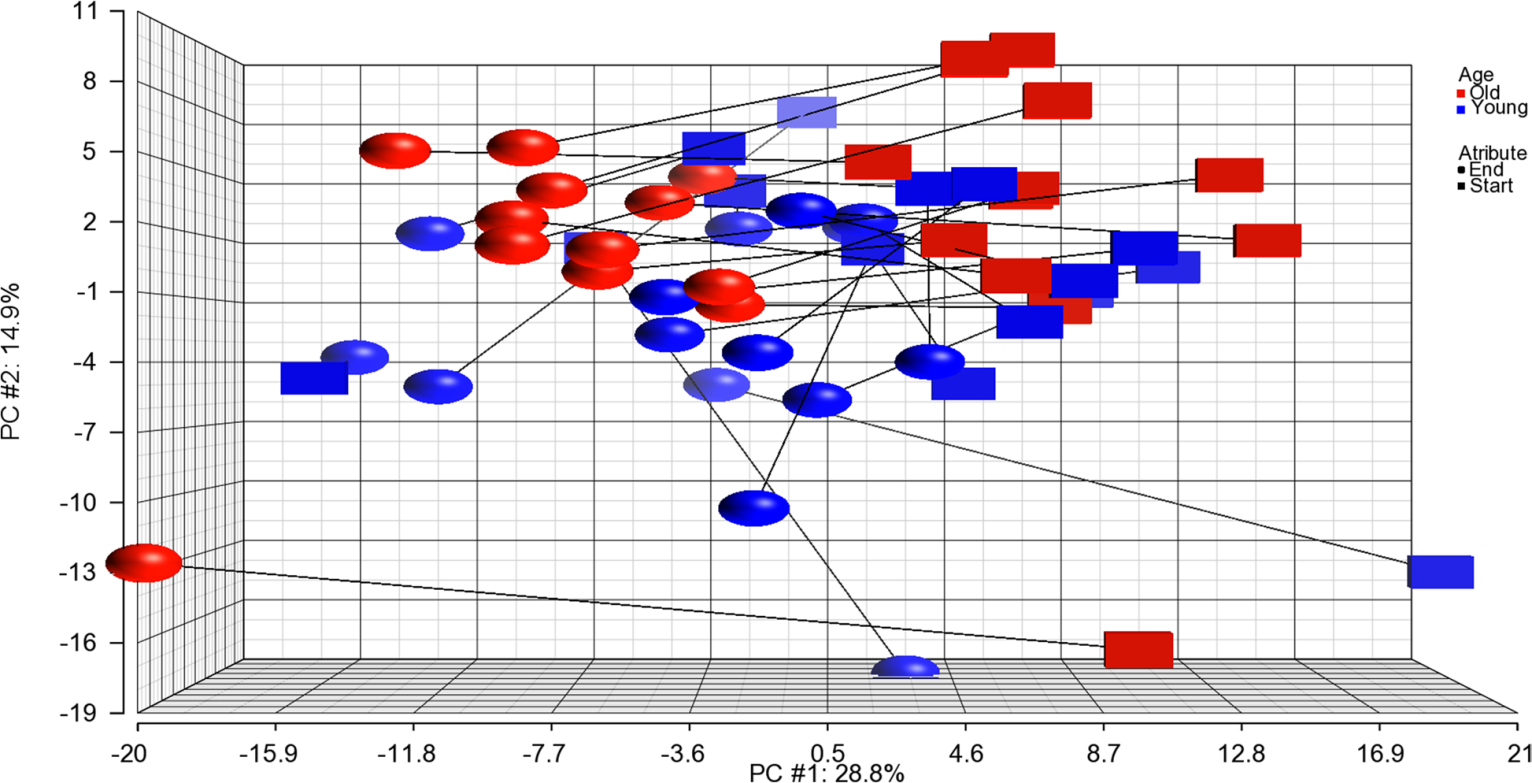
PCA of young and old in BSU cohort, before and after exercise. Principal component analysis of the 249 common transcripts changed in old Oslo and old BSU cohorts at q-value < 0.1 (Table S1). The plot includes those transcripts analyzed in old people (red color) or young people (blue color) of the BSU cohorts before (squares) and after (balls) the strength training period. Each symbol represents one person depicted as one average transcript signal value of the 249 transcripts (Table S1). The main differences in gene expression are located along the x-axis while minor differences are located along the y- and z-axes. A faint colour indicates that the symbol is placed somewhat back along the Z axis

To illustrate the changes in young vs old, we subjected the 249 transcripts in the BSU young and old cohorts to principal component analysis (PCA) (Fig. 3). The figure demonstrates that for all participants, with the exception of a few young persons, expression changes were in the same direction (leftwards). Furthermore, visual inspection indicates that the old cohorts are differentially grouped as compared to the young.

### Comparison of the basal iliac and thigh muscle transcriptomes

To test if the basal transcriptome of a given skeletal muscle is representative for other muscles and thereby assess the general validity of the transcriptome data, we compared the iliac musculature from 24 age- and BMI-matched PM women (validation group) with the results from the 35 thigh muscle donors before intervention (Table 1). The transcriptomes did not differ statistically but showed a major degree of similarity (students t-test *p*-value = 0.985 and Pearson correlation coefficient of *r* = 0.78 (*p* < 0.0001)) (Fig. S1). Thus, the non-exercised skeletal musculatures from different anatomical sites have a common transcriptional background from which training initiates gene activity changes.

### Functional enrichment analyses

The 249 common genes changing expression in the Oslo and old BSU cohorts were subjected to IPA analysis to identify affected biological processes. For comparison, the 78 genes that changed expression in the young BSU cohort (at q-value < 0.2) were analyzed similarly. Table 2 demonstrates that several functions associated with a healthy outcome, especially related to the vascular system, were increased (red color) in the old following strength training. The genes from our dataset involved in the topmost activated function “Vasculogenesis” were presented in Fig. S2. Functions generally associated with a negative health outcome were decreased (blue color), such as “glucose metabolism disorder”,”congenital anomaly of cardiovascular system and disorder of blood pressure”. Among the functions systems more intuitively associated with maximal strength, only “muscle contraction” is increased. In the young, only two categories reached significance, including “Migration of cells” and “Hydrolysis of phospholipid.”

### SNPs in muscle eQTLs associated with training-responsive muscle transcripts and correlated to loci for handgrip strength

Several studies have shown that handgrip strength reflects general muscle strength and sarcopenia [15, 16], but a large, well-powered GWAS of resistance exercise training does not exist. Thus, we hypothesized that genes that respond well to resistance training could overlap those associated with handgrip strength. Using candidate genes associated with handgrip strength obtained from a large genome-wide meta-analysis [11] we looked for overlapping transcripts also responsive to long-duration heavy-load exercise. As described in the Methods, we first used established muscle eQTL databases to identify SNPs known to affect the expression of 249 genes (Table S1) at near suggestive genome significance. Then, we tested these SNPs for association with low grip strength/weakness phenotype in females, and identified 26 genes (Table 3).

**Table 3:** SNPs in skeletal muscle eQTLs associated with training-responsive genes and handgrip strength

**Training-associated gene**		**SNP**	**Variant ID**	**eQTL p-values for skeletal muscle expression**	**eQTL *p*-values for handgrip strength**
*HSPG2*	Heparan Sulfate Proteoglycan 2	rs71636995	chr1_21877520_C_T_b38	1.40E-05	3.87E-03
*CYP4B1*	Cytochrome P450 Family 4 Subfamily B Member 1	rs11211375	chr1_46836964_A_G_b38	9.20E-20	3.50E-03
*HIBCH*	3-Hydroxyisobutyryl-CoA Hydrolase	rs201160834	chr2_190304632_A_AT_b38	3.40E-08	2.04E-03
*RFTN2*	Raftlin Family Member 2	rs68022726	chr2_197566490_A_G_b38	5.60E-05	6.69E-03
*DGKD*	Diacylglycerol Kinase Delta	rs76145205	chr2_233584763_G_T_b38	4.40E-06	1.99E-03
*SCAP*	SREBF Chaperone	rs62260743	chr3_47541718_C_T_b38	7.00E-05	7.18E-03
*NOA1*	Nitric Oxide Associated 1	rs1277271	chr4_56971181_T_C_b38	1.30E-08	2.12E-03
*DHFR*	Dihydrofolate Reductase	rs26266	chr5_80744875_T_C_b38	3.70E-41	6.05E-03
*NQO2*	N-Ribosyldihydronicotinamide: Quinone Reductase 2	rs28383595	chr6_3001234_T_C_b38	2.50E-08	5.97E-03
*NOTCH4*	Notch Receptor 4	rs1281934	chr6_32616605_A_G_b38	9.70E-08	3.78E-08
*CSGALNACT1*	Chondroitin Sulfate N-Acetylgalactosaminyltransferase 1	rs11204056	chr8_19596883_C_T_b38	4.70E-11	9.93E-03
*ANK1*	Ankyrin 1	rs62508166	chr8_41679800_G_A_b38	4.20E-09	8.91E-03
*COL15A1*	Collagen Type XV Alpha 1 Chain	rs7026938	chr9_98875916_C_T_b38	2.80E-05	9.15E-03
*TRPT1*	TRNA Phosphotransferase 1	rs72924202	chr11_64365756_C_T_b38	1.30E-18	1.31E-03
*FZD4*	Frizzled Class Receptor 4	rs611538	chr11_87125491_T_G_b38	5.30E-07	7.85E-03
*CEP57*	Centrosomal Protein 57	rs571413	chr11_95793424_C_T_b38	1.30E-14	2.03E-03
*STX2*	Syntaxin 2	rs7137314	chr12_130756804_C_T_b38	3.80E-12	7.86E-03
*TMTC1*	Transmembrane O-Mannosyltransferase Targeting Cadherins 1	rs11050591	chr12_29971149_C_A_b38	1.50E-06	8.41E-03
*RCBTB2*	RCC1 And BTB Domain Containing Protein 2	rs7991892	chr13_47942622_C_T_b38	6.80E-05	1.60E-03
*PPP1R15A*	Protein Phosphatase 1 Regulatory Subunit 15A	rs4802517	chr19_48878914_T_C_b38	3.50E-05	7.90E-03
*ACSS1*	Acyl-CoA Synthetase Short Chain Family Member 1	rs6049932	chr20_24738298_C_G_b38	3.90E-06	6.57E-03
*GSS*	Glutathione Synthetase	rs3891369	chr20_34573323_C_T_b38	7.70E-07	6.66E-06
*ACSS2*	Acyl-CoA Synthetase Short Chain Family Member 2	rs6058105	chr20_34683915_C_T_b38	3.60E-08	1.63E-06
*JPH2*	Junctophilin 2	rs77792994	chr20_44130642_G_A_b38	9.40E-05	9.23E-03
*MICAL3*	Microtubule Associated Monooxygenase, Calponin And LIM Domain Containing 3	rs56076143	chr22_17898151_C_T_b38	5.70E-05	2.18E-03
*MAPK12*	Mitogen-Activated Protein Kinase 12	rs2294394	chr22_50071829_T_C_b38	1.10E-05	6.61E-04

Variant ID refers to chromosome_position_ref_alt_build (*ref* reference nucleotide, *alt* alternative nucleotide, *build* genome build version)

## Discussion

The core of the present study comprises a well-characterized group of PM women participating in a heavy-load exercise program and is the largest uniform group published so far aiming to describe in-depth the global engagement of training-sensitive genes in older adults.

During prolonged heavy-load exercise in PM women, the major groups of training-sensitive genes as judged by q-values or fold-change, encode extracellular matrix exposed receptors and adhesion proteins (Table S1). The importance of signal pathways through receptor activation and growth factors are recognized, e.g., IGF1, which initiates myotube hypertrophy via the PI(3)K–Akt–mTOR and PI(3)K–Akt–GSK pathways [17].

Topmost over-represented diseases and functions relate to circulation, focusing on development of new vasculature e.g. “Vasculogenesis” and”Angiogenesis” (Table 2), mediated by functions such as “proliferation of vascular cells”, “Cell movement of smooth muscle cells”, “Tubulations of cells” and “transmigration of cells”. These are in turn promoted by factors/processes like hypoxia, shear stress, adenine and muscle stretch, leading to increased expression of key regulatory genes, such as vascular endothelial growth factor (*VEGF*) and matrix metalloproteinases (*MMP*s) [18].

Studies have shown that mitochondrial function and genesis are stimulated during long term RET [19–21], and it is generally accepted that the enhancement of oxidative phosphorylation generating ATP is a prerequisite for successful strength training [19]. This study shows no over-representation of functional pathways related to increased mitochondrial activity, perhaps due to lack of statistical power or insufficient length of the intervention. Still, several genes encoding proteins known to induce mitochondrial biogenesis have been shown to increase expression after exercise, e.g., *CAMK2A* and *CAMK2B* (Table S1) [22, 23]. A recent study indicates that IGF2 also promotes mitochondrial biogenesis in skeletal muscle [24], and similar results have been obtained for IGF1 [25–27], and both transcripts increase after exercise (Table S1). Although pathways for mitochondrial biogenesis were not over-represented after the intervention, those involved in vasculogenesis and angiogenesis were highly upregulated with resistance training. These changes probably reflect increased capillarization as observed in a similar heavy-load resistance training intervention in older men [28], confirming that resistance training can induce aerobic adaptations especially in older deconditioned muscles. Furthermore, it has been suggested that increased capillarization is critical for the hypertrophic response to resistance training [29]. Consequently, the observed upregulation of pathways involved in vasculogenesis and angiogenesis may therefore be a critical part of the normal muscle growth induced by heavy-load resistance training.

A recent genome-wide association meta-analysis studying the handgrip phenotype and involving more than 250,000 Europeans of both sexes aged ≥ 60 years presents 15 loci associated with muscle weakness [11]. We used their data to analyze whether some of the handgrip-associated loci also relate to the genes responsive for heavy-load exercise after limiting the search to candidate muscle eQTLs. Twenty-six transcripts correlate to SNPs in muscle eQTLs and handgrip (Table 3). Of these, *CYP4B1*, encoding a member of the cytochrome 450 superfamily of enzymes, shows the highest correlation to muscle eQTLs, whereas *NOTCH4* (Notch receptor 4) and *GSS* (glutathione synthetase) are the most significantly associated with handgrip eQTLs. GSS protects against oxidative damage [30] and is thus of obvious importance for muscle reconstitution after stress and thereby also muscle strength and now a possible genetic marker of sarcopenia. Drugs that increase the activity of GSS could be a target for treatment of extreme muscle weakness. In a recent analysis of FDA-approved drugs 33 of the 50 approved in 2021 (66%) were supported by genetic evidence for the gene encoding the target of the drug [31]. Thus, the genes we report that are supported by evidence from muscle eQTL data and genetic associations with weakness (low hand grip strength) in older adults are the most likely to have a causal role in response to exercise and represent possible future intervention targets.

The reproducibility of previous studies in terms of finding common exercise sensitive genes in old vs young is surprisingly low: When comparing genes that are found changed in young vs old in three datasets [9, 32, 33] only one common gene is detected for the three studies and less than 30 were common between any two of these three studies [33]. Relatively few transcripts are changed in the BSU cohort of young (78 at FDR < 0.2) and only two items were identified under”Diseases and Functions” (Table 2), maybe due to a smaller, more heterogeneous cohort of younger premenopausal women and men, possibly starting at a “higher” functional level. The number of genes that are affected by training is much higher in the older BSU adults (average age 84 years) than in younger (average age 24 years) BSU participants and also higher when compared to the Oslo cohort (average age 70 years) (Table 1). Combined, these observations support that RET has a stronger transcriptional impact in older adults, and a clear noticeable difference is observed in the two elderly age groups of 14 years difference. This conclusion confirms the results of Raue et al. [9] identifying a higher number of genes that were activated in older adults as compared to younger in study groups of 12 and 15 persons, respectively. The present study, taking advantage of two larger cohorts, is able to markedly expand the number of affected genes in older adults from 144 to 249 thereby also allowing for improved functional enrichment analyses (IPA). *Strengths and limitations of the study.* As main strength of the study, we present the largest uniform group of participants in a RET study to date and include analysis of an independent group for replication. In addition, these molecular changes are firmly anchored in prior reported physiological results[12] showing improvement of several parameters (muscle mass, mechanical strength and balance improvement). The inclusion of two elderly groups allows us to suggest that our ability to respond positively to physical training as reflected in advantageous muscle gene expression, is a quality lasting throughout life.

Methodologically, the study is sound as the transcript microarray data from both sets of musculatures have been technically validated using RT-PCR, with satisfactory results. Weak sides are related to limited number of participants, especially in the BSU cohort of young. Also, the cohort of younger premenopausal women and men shows higher transcript heterogeneity, and they may also have started at a “higher” functional level which will reduce the probability to find small and moderate differences caused by the intervention. A relative limitation includes different average age of the Oslo and the older BSU cohorts, possibly leading to a reduced number of detected RET responsive genes. Furthermore, the number of identified RET responsive muscle genes would probably be higher with a larger replication cohort, allowing for stronger statistics. Also, the Oslo group followed a whole-body approach while the BSU cohort was focused on leg exercises, which may have affected the results somewhat. The Oslo cohort appears to be more ethnically uniform than the BSU cohort, and lack of homogeneity may represent a possibly negative aspect and lead to fewer genes discovered. However, the somewhat heterogeneous cohorts may make the results representative for a wider population. The Oslo cohort study comprises only older women, while older men and women in the BSU cohort are pooled to increase statistical power. Again, this may be considered negative with respect to number of identified affected genes, but on the other hand, the results may have relevance for both sexes.

When comparing thigh and iliac muscle transcriptomes, we used different array types for the two muscle types. Thus, if the same array type had been used, we consider it likely that similarity would be even higher than the calculated *r*-value of 0.78 (Fig. S1).

We identified 249 training sensitive, common genes adapting data from two studies, and their functions converged towards vasculogenesis, improvement of circulation and blood pressure regulation in addition to counterbalancing unhealthy aspects of metabolism. As muscular asthenia, weakness, and sarcopenia affect ageing women and men in general, it is also likely that the results are valid for all people. Some of the genes identified may lend themselves to possible drug targets for reducing severe muscle weakness.

## Conclusions

The largest uniform cohort of older adults, clinically well characterized females transcriptionally profiled, yielded 491 (q-value < 0.1) training sensitive genes after prolonged RET exercise. These results were validated in an independent cohort of older men and women detecting 249 (q-value < 0.1) annotated common transcripts. Using IPA for gene enrichment, these transcripts predicted increase in several aspects of vascularization and muscle contractions, whereas impairment of “Glucose metabolism” and blood pressure regulation was reduced. Similar effects were not observed in a trained cohort of young persons. The results identify the underlying molecular mechanisms bridging the improved mechanical muscle performance obtained during long-term heavy-load strength training in older adults.

## Abbreviations and explanations

**Table:** 

Abbreviation	Term	Brief explanation
BMI	Body Mass Index	BMI = kg/m^2^ where kg is a person's weight in kilograms and m^2^ is their height in meters squared
eQTL	Expressed quantitative loci	Genomic loci (with DNA sequence/nucleotide variants) that explain variation in expression levels of mRNAs
GEO	Gene Expression Omnibus	International public repository that archives and freely distributes high-throughput gene expression and other functional genomics data sets
	Genome	All genetic information of an organism
GWAS	Genome-wide association studies	Observational studies of a genome-wide set of genetic variants (normally SNPs) in different individuals to see if any variant is associated with a trait
GTEx portal	Genotype-Tissue Expression portal	A public database of identified genetic variants (eQTL) that influence how genes are turned on and off in human tissues and organs
IPA	Ingenuity Pathway Analysis	Commercial web-based software application for pathway analysis using a manually curated knowledge base
PCA	principal component analysis	A statistical procedure that allows you to summarize the information content in large data tables by means of a smaller set of “summary indices” that can be more easily visualized and analyzed
RM	Repetition Maximum	The most weight that can be lifted for a defined number of exercise movements
RET	Resistance-Exercise Training	Exercise that causes the muscles to contract against an external resistance with the expectation of increases in strength, power, hypertrophy, and/or endurance
RT-PCR	Reverse transcription Polymerase Chain Reaction (PCR)	Laboratory technique combining reverse transcription of RNA into complementary DNA (cDNA) and amplification of specific DNA targets using PCR. Primarily used to measure amounts of specific RNAs
SNPs	Single Nucleotide Polymorphisms	Single nucleotide variants at specific positions in the genome
	Transcriptome	The set of all RNA transcripts, including coding and non-coding, in an individual or a population of cells

## Supplementary Information

**Addiitional file 1: Figure S1.** Comparison of transcript signal levels between os ilium–associated muscle and thigh muscle. **Figure S2.** Genes within the Diseases and Function category Vasculogenesis with increased (red) or reduced (green) expression end vs start of the training period in elderly as compared to young. **Table S1.** Common annotated genes changed in elderly in both Oslo and BSU cohorts at Q-value<0.1.

## Data Availability

Microarray data from the BSU cohort have been submitted to the GEO database (www.ncbi.nlm.nih.gov/geo/): GSE28422, GSE28392, and GSE25941. Microarray data from the Oslo cohort is available on request.

## References

[1] Rizzoli R, Reginster JY, Arnal JF, Bautmans I, Beaudart C, Bischoff-Ferrari H, Biver E, Boonen S, Brandi ML, Chines A, Cooper C, Epstein S, Fielding RA, Goodpaster B, Kanis JA, Kaufman JM, Laslop A, Malafarina V, Manas LR, Mitlak BH, Oreffo RO, Petermans J, Reid K, Rolland Y, Sayer AA, Tsouderos Y, Visser M, Bruyere O (2013). Quality of life in sarcopenia and frailty. Calcif Tissue Int.

[2] Goates S, Du K, Arensberg MB, Gaillard T, Guralnik J, Pereira SL (2019). Economic impact of hospitalizations in US adults with sarcopenia. J Frailty Aging.

[3] C. Nordic Burden of Disease (2019). Life expectancy and disease burden in the nordic countries: results from the global burden of diseases, injuries, and risk factors study 2017. Lancet Public Health.

[4] Jiahao L, Jiajin L, Yifan L (2021). Effects of resistance training on insulin sensitivity in the elderly: a meta-analysis of randomized controlled trials. J Exerc Sci Fit.

[5] Grgic J, Garofolini A, Orazem J, Sabol F, Schoenfeld BJ, Pedisic Z (2020). Effects of resistance training on muscle size and strength in very elderly adults: a systematic review and meta-analysis of randomized controlled trials. Sports Med.

[6] Ramos-Campo DJ, Andreu-Caravaca L, Carrasco-Poyatos M, Benito PJ, Rubio-Arias JA. Effects of circuit resistance training on body composition, strength, and cardiorespiratory fitness in middle-aged and older women: a systematic review and meta-analysis. J Aging Phys Act. 2021;30:1–14. 10.1123/japa.2021-0204 34627129

[7] Pillon NJ, Gabriel BM, Dollet L, Smith JAB, SardonPuig L, Botella J, Bishop DJ, Krook A, Zierath JR (2020). Transcriptomic profiling of skeletal muscle adaptations to exercise and inactivity. Nat Commun.

[8] Lavin KM, Bell MB, McAdam JS, Peck BD, Walton RG, Windham ST, Tuggle SC, Long DE, Kern PA, Peterson CA, Bamman MM (2021). Muscle transcriptional networks linked to resistance exercise training hypertrophic response heterogeneity. Physiol Genomics.

[9] Raue U, Trappe TA, Estrem ST, Qian HR, Helvering LM, Smith RC, Trappe S (2012). Transcriptome signature of resistance exercise adaptations: mixed muscle and fiber type specific profiles in young and old adults. J Appl Physiol.

[10] Olstad OK, Gautvik VT, LeBlanc M, Kvernevik KJ, Utheim TP, Runningen A, Wiig H, Kirkegaard C, Raastad T, Reppe S, Gautvik KM. Postmenopausal osteoporosis is a musculoskeletal disease with a common genetic trait which responds to strength training: a translational intervention study. Ther Adv Musculoskelet Dis In press. 2020;12:1759720X20929443. 10.1177/1759720X20929443 PMC7268165 32536985

[11] Jones G, Trajanoska K, Santanasto AJ, Stringa N, Kuo CL, Atkins JL, Lewis JR, Duong T, Hong S, Biggs ML, Luan J, Sarnowski C, Lunetta KL, Tanaka T, Wojczynski MK, Cvejkus R, Nethander M, Ghasemi S, Yang J, Zillikens MC, Walter S, Sicinski K, Kague E, Ackert-Bicknell CL, Arking DE, Windham BG, Boerwinkle E, Grove ML, Graff M, Spira D, Demuth I, van der Velde N, de Groot L, Psaty BM, Odden MC, Fohner AE, Langenberg C, Wareham NJ, Bandinelli S, van Schoor NM, Huisman M, Tan Q, Zmuda J, Mellstrom D, Karlsson M, Bennett DA, Buchman AS, De Jager PL, Uitterlinden AG, Volker U, Kocher T, Teumer A, Rodriguez-Manas L, Garcia FJ, Carnicero JA, Herd P, Bertram L, Ohlsson C, Murabito JM, Melzer D, Kuchel GA, Ferrucci L, Karasik D, Rivadeneira F, Kiel DP, Pilling LC (2021). Genome-wide meta-analysis of muscle weakness identifies 15 susceptibility loci in older men and women. Nat Commun.

[12] Raastad T, Kvernevik KJ, Johansen M, Runningen A, Dullerud R, Kvamme N, Solberg P, Gautvik KM (2015). Marked Improvement in Physical Function through Gains in Muscle Strength and Thigh Muscle Size after Heavy-Load Strength Training in Women with Established Postmenopausal Osteoporosis. J Osteopor Phys Act.

[13] Reppe S, Refvem H, Gautvik VT, Olstad OK, Hovring PI, Reinholt FP, Holden M, Frigessi A, Jemtland R, Gautvik KM (2010). Eight genes are highly associated with BMD variation in postmenopausal Caucasian women. Bone.

[14] Buyers P, Smith R (1967). Trephine for full-thickness iliac-crest biopsy. Br Med J.

[15] Fonseca J, Machado FVC, Santin LC, Andrello AC, Schneider LP, Fernandes Belo L, Rodrigues A, FernandesRugila D, Furlanetto KC, Hernandes NA, Pitta F (2021). Handgrip strength as a reflection of general muscle strength in chronic obstructive pulmonary disease. COPD.

[16] Fragala MS, Alley DE, Shardell MD, Harris TB, McLean RR, Kiel DP, Cawthon PM, Dam TT, Ferrucci L, Guralnik JM, Kritchevsky SB, Vassileva MT, Gudnason V, Eiriksdottir G, Koster A, Newman A, Siggeirsdottir K, Satterfield S, Studenski SA, Kenny AM (2016). Comparison of handgrip and leg extension strength in predicting slow gait speed in older adults. J Am Geriatr Soc.

[17] Rommel C, Bodine SC, Clarke BA, Rossman R, Nunez L, Stitt TN, Yancopoulos GD, Glass DJ (2001). Mediation of IGF-1-induced skeletal myotube hypertrophy by PI(3)K/Akt/mTOR and PI(3)K/Akt/GSK3 pathways. Nat Cell Biol.

[18] Ranjbar K, Fayazi B. Vascularisation of Skeletal Muscle. In: Valarmathi MT, editor. Muscle Cells - Recent Advances and Future Perspectives. IntechOpen Book Series; 2020. 1. 10.5772/intechopen.77689. ISBN978-1-78923-968-3. https://www.intechopen.com/books/7870.

[19] Groennebaek T, Vissing K (2017). Impact of Resistance Training on Skeletal Muscle Mitochondrial Biogenesis, Content, and Function. Front Physiol.

[20] Grevendonk L, Connell NJ, McCrum C, Fealy CE, Bilet L, Bruls YMH, Mevenkamp J, Schrauwen-Hinderling VB, Jorgensen JA, Moonen-Kornips E, Schaart G, Havekes B, de Vogel-van J, den Bosch MCE, Bragt K, Meijer P, Schrauwen JH (2021). Impact of aging and exercise on skeletal muscle mitochondrial capacity, energy metabolism, and physical function. Nat Commun.

[21] Porter C, Reidy PT, Bhattarai N, Sidossis LS, Rasmussen BB (2015). Resistance exercise training alters mitochondrial function in human skeletal muscle. Med Sci Sports Exerc.

[22] Holloszy JO (2011). Regulation of mitochondrial biogenesis and GLUT4 expression by exercise. Compr Physiol.

[23] Memme JM, Hood DA (2020). Molecular Basis for the Therapeutic Effects of Exercise on Mitochondrial Defects. Front Physiol.

[24] Zhu Y, Gui W, Tan B, Du Y, Zhou J, Wu F, Li H, Lin X (2021). IGF2 deficiency causes mitochondrial defects in skeletal muscle. Clin Sci (Lond).

[25] Lyons A, Coleman M, Riis S, Favre C, O'Flanagan CH, Zhdanov AV, Papkovsky DB, Hursting SD, O'Connor R (2017). Insulin-like growth factor 1 signaling is essential for mitochondrial biogenesis and mitophagy in cancer cells. J Biol Chem.

[26] Hou X, Li Z, Higashi Y, Delafontaine P, Sukhanov S (2020). Insulin-Like Growth Factor I Prevents Cellular Aging via Activation of Mitophagy. J Aging Res.

[27] Riis S, Murray JB, O'Connor R (2020). IGF-1 Signalling Regulates Mitochondria Dynamics and Turnover through a Conserved GSK-3beta-Nrf2-BNIP3 Pathway. Cells.

[28] Verdijk LB, Snijders T, Holloway TM, Kranenburg JVAN (2016). LCCVAN Loon, Resistance Training Increases Skeletal Muscle Capillarization in Healthy Older Men. Med Sci Sports Exerc.

[29] Snijders T, Nederveen JP, Joanisse S, Leenders M, Verdijk LB, van Loon LJ, Parise G (2017). Muscle fibre capillarization is a critical factor in muscle fibre hypertrophy during resistance exercise training in older men. J Cachexia Sarcopenia Muscle.

[30] Njalsson R (2005). Glutathione synthetase deficiency. Cell Mol Life Sci.

[31] Mullard A (2022). 2021 FDA approvals. Nat Rev Drug Discov.

[32] Melov S, Tarnopolsky MA, Beckman K, Felkey K, Hubbard A (2007). Resistance exercise reverses aging in human skeletal muscle. PLoS ONE.

[33] Phillips BE, Williams JP, Gustafsson T, Bouchard C, Rankinen T, Knudsen S, Smith K, Timmons JA, Atherton PJ (2013). Molecular networks of human muscle adaptation to exercise and age. PLoS Genet.

