# Supplementary material for: Heavy-load exercise in older adults activates vasculogenesis and has a stronger impact on muscle gene expression than in young adults
Source: Eur Rev Aging Phys Act. 2022 Oct 1;19:23. doi: 10.1186/s11556-022-00304-1 (PMC9526277; doi:10.1186/s11556-022-00304-1)
Supplement: Supplementary file 1 — Addiitional file 1: Figure S1. Comparison of transcript signal levels between os ilium–associated muscle and thigh muscle. Figure S2. Genes within the Diseases and Function category Vasculogenesis with increased (red) or reduced (green) expression end vs start of the training period in elderly as compared to young. Table S1. Common annotated genes changed in elderly in both Oslo and BSU cohorts at Q-value<0.1. [file 11556_2022_304_MOESM1_ESM.pdf]

SUPPLEMENTARY

Heavy-load exercise in older adults activates vasculogenesis and has a stronger impact on muscle gene expression than in young adults

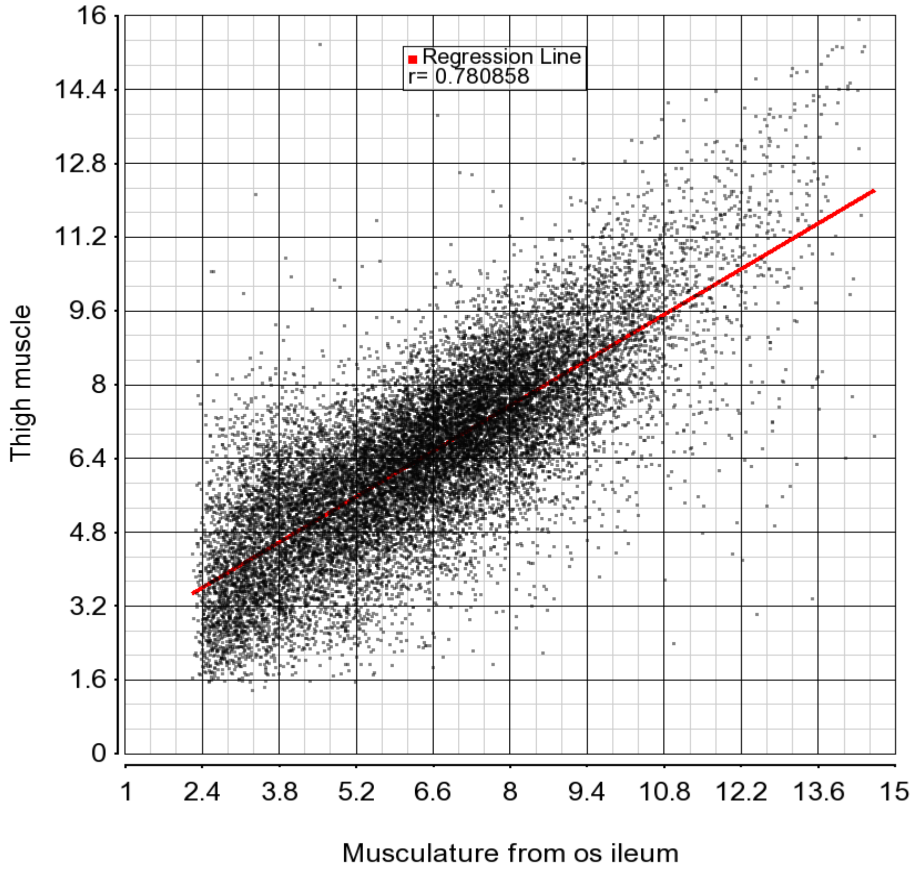

Figure S1. Comparison of transcript signal levels between os ilium–associated muscle and thigh muscle. Numbers on the axes represent log_2 transformed signal levels. For technical reasons, it was possible to use 17,652 transcripts or 93.5% in further statistical analysis, which is presented in the scatterplot.

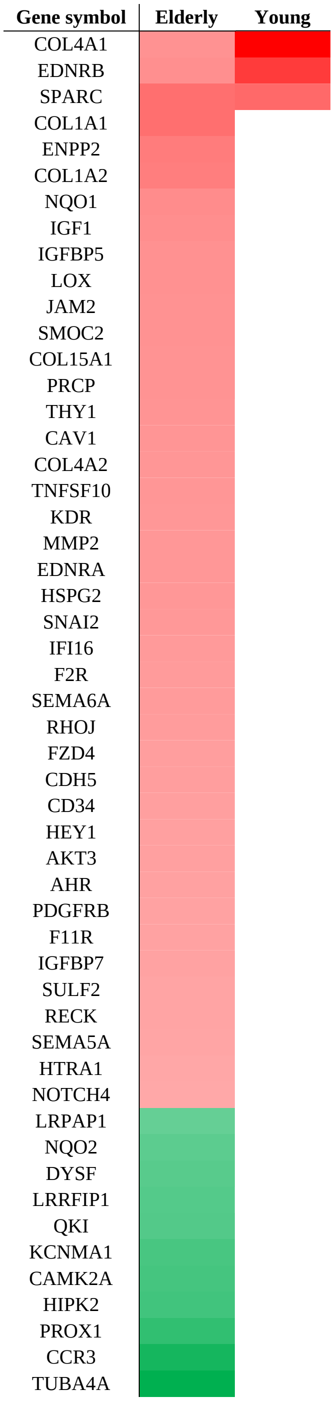

Figure S2. Genes within the Diseases and Function category Vasculogenesis with increased (red) or reduced (green) expression end vs start of the training period in elderly as compared to young.

Table S1. Common annotated genes changed in elderly in both Oslo and BSU cohorts at Q-value<0.1

		Oslo cohort			BSU cohort		
**Gene Symbol**	**Gene Title**	**Fold-Change (End vs. Start)**	**p-value (End vs. Start)**	**Qvalue (End vs. Start)**	**Fold-Change (End vs. Start)**	**p-value (End vs. Start)**	**Qvalue (End vs. Start)**
**ABCG1**	ATP-binding cassette, sub-family G (WHITE), member 1	1.11	6.0E-04	4.0E-02	1.16	2.00E-02	8.14E-02
**ACSS1**	acyl-CoA synthetase short-chain family member 1	-1.12	2.8E-03	8.4E-02	-1.25	1.41E-02	6.91E-02
**ACSS2**	acyl-CoA synthetase short-chain family member 2	-1.12	2.3E-04	2.9E-02	-1.18	1.61E-04	8.02E-03
**ACTN2**	actinin, alpha 2	-1.09	1.7E-05	1.1E-02	-1.10	2.34E-03	2.92E-02
**ADPRHL1**	ADP-ribosylhydrolase like 1	-1.16	4.2E-05	1.4E-02	-1.18	4.25E-03	3.84E-02
**AHR**	aryl hydrocarbon receptor	1.11	1.8E-03	6.4E-02	1.37	1.08E-04	6.71E-03
**AKT3**	v-akt murine thymoma viral oncogene homolog 3	1.12	2.8E-03	8.4E-02	1.23	2.24E-03	2.85E-02
**ALPK3**	alpha-kinase 3	-1.16	4.5E-04	3.6E-02	-1.31	6.68E-04	1.56E-02
**ALYREF**	Aly/REF export factor	-1.09	3.9E-04	3.5E-02	-1.21	2.32E-03	2.91E-02
**ANK1**	ankyrin 1, erythrocytic	-1.08	2.0E-03	6.8E-02	-1.25	5.13E-03	4.23E-02
**ANKRD2**	ankyrin repeat domain 2 (stretch responsive muscle)	-1.47	6.3E-04	4.0E-02	-1.82	6.65E-04	1.55E-02
**ANXA11**	annexin A11	-1.10	1.2E-03	5.4E-02	-1.14	6.56E-03	4.81E-02
**AP1B1**	adaptor-related protein complex 1, beta 1 subunit	-1.06	8.0E-04	4.4E-02	-1.12	1.05E-02	6.01E-02
**APRT**	adenine phosphoribosyltransferase	-1.08	4.3E-04	3.6E-02	-1.11	1.30E-02	6.65E-02
**ASPN**	asporin	1.47	1.0E-04	2.1E-02	1.92	2.25E-03	2.86E-02
**AVPR1A**	arginine vasopressin receptor 1A	1.14	5.7E-05	1.6E-02	1.76	1.15E-04	6.94E-03
**B3GALTL**	beta 1,3-galactosyltransferase-like	1.14	1.3E-03	5.7E-02	1.34	4.77E-04	1.34E-02
**BACE2**	beta-site APP-cleaving enzyme 2	1.08	2.1E-03	7.0E-02	1.38	3.29E-04	1.15E-02
**BNIP3L**	BCL2/adenovirus E1B 19kDa interacting protein 3-like	1.08	3.7E-03	9.5E-02	1.15	2.79E-02	9.49E-02
**BOP1**	block of proliferation 1 /// microRNA 7112	-1.06	1.1E-03	5.2E-02	-1.22	9.31E-04	1.86E-02
**C20orf166**	chromosome 20 open reading frame 166	-1.21	5.4E-05	1.6E-02	-1.13	5.06E-03	4.21E-02
**CAMK2A**	calcium/calmodulin-dependent protein kinase II alpha	-1.14	6.4E-04	4.0E-02	-1.20	3.88E-03	3.67E-02
**CAMK2B**	calcium/calmodulin-dependent protein kinase II beta	-1.11	9.2E-05	1.9E-02	-1.23	5.12E-04	1.38E-02
**CAPN6**	calpain 6	1.19	3.5E-03	9.3E-02	1.64	1.05E-03	1.98E-02
**CARM1**	coactivator-associated arginine methyltransferase 1	-1.07	3.7E-03	9.7E-02	-1.12	2.53E-02	9.04E-02
**CASP4**	caspase 4, apoptosis-related cysteine peptidase	1.14	1.5E-03	6.2E-02	1.38	1.18E-02	6.36E-02
**CAV1**	caveolin 1, caveolae protein, 22kDa	1.16	3.7E-04	3.5E-02	1.33	1.50E-05	2.74E-03
**CCR3**	chemokine (C-C motif) receptor 3	-1.25	2.4E-04	2.9E-02	-1.43	4.26E-03	3.84E-02
**CD34**	CD34 molecule	1.12	2.6E-03	8.0E-02	1.35	3.48E-06	1.51E-03
**CD93**	CD93 molecule	1.12	2.2E-03	7.2E-02	1.70	5.54E-08	1.77E-04
**CDC42EP4**	CDC42 effector protein (Rho GTPase binding) 4	-1.07	2.0E-03	6.8E-02	-1.18	1.10E-02	6.13E-02
**CDH5**	cadherin 5, type 2 (vascular endothelium)	1.12	1.7E-03	6.3E-02	1.58	9.52E-07	8.66E-04
**CDH6**	cadherin 6, type 2, K-cadherin (fetal kidney)	1.11	2.2E-03	7.1E-02	1.62	2.63E-05	3.57E-03
**CEP57**	centrosomal protein 57kDa	1.10	5.2E-04	3.8E-02	1.19	2.22E-02	8.52E-02
**CHRDL1**	chordin-like 1	1.19	1.2E-03	5.3E-02	1.39	1.45E-02	7.01E-02
**CLEC14A**	C-type lectin domain family 14, member A	1.08	2.0E-03	6.8E-02	1.29	2.06E-04	9.20E-03
**CLEC2D**	C-type lectin domain family 2, member D	1.10	1.4E-03	5.7E-02	1.42	1.43E-02	6.96E-02
**CLTCL1**	clathrin, heavy chain-like 1	-1.10	1.9E-03	6.6E-02	-1.24	6.31E-05	5.24E-03
**CMIP**	c-Maf inducing protein	1.09	1.8E-03	6.4E-02	1.27	2.61E-02	9.17E-02
**CNNM4**	cyclin M4	-1.18	5.8E-05	1.6E-02	-1.24	9.63E-05	6.37E-03
**COL15A1**	collagen, type XV, alpha 1	1.17	3.4E-05	1.2E-02	1.40	3.67E-04	1.20E-02
**COL1A1**	collagen, type I, alpha 1	1.33	3.6E-03	9.5E-02	1.94	5.61E-04	1.44E-02
**COL1A2**	collagen, type I, alpha 2	1.26	1.1E-03	5.1E-02	1.57	3.39E-04	1.16E-02
**COL3A1**	collagen, type III, alpha 1	1.35	6.2E-04	4.0E-02	1.69	3.30E-05	4.02E-03
**COL4A1**	collagen, type IV, alpha 1	1.18	6.3E-05	1.6E-02	2.45	5.72E-08	1.77E-04
**COL4A2**	collagen, type IV, alpha 2	1.16	2.0E-05	1.1E-02	2.31	6.02E-07	6.76E-04
**COL5A2**	collagen, type V, alpha 2	1.18	3.6E-03	9.4E-02	1.87	1.38E-03	2.22E-02
**COL6A3**	collagen, type VI, alpha 3	1.17	2.8E-03	8.4E-02	1.24	3.28E-03	3.43E-02
**COLEC12**	collectin sub-family member 12	1.18	2.2E-05	1.1E-02	1.30	2.62E-03	3.07E-02
**CSGALNACT1**	chondroitin sulfate N-acetylgalactosaminyltransferase 1	1.12	2.5E-03	7.8E-02	1.60	8.58E-05	6.06E-03
**CTSK**	cathepsin K	1.17	2.5E-03	7.8E-02	1.32	1.41E-02	6.91E-02
**CTSO**	cathepsin O	1.12	6.2E-04	4.0E-02	1.23	2.26E-02	8.60E-02
**CYB5D2**	cytochrome b5 domain containing 2	1.07	1.0E-03	5.0E-02	1.13	4.10E-03	3.77E-02
**CYP4B1**	cytochrome P450, family 4, subfamily B, polypeptide 1	-1.24	1.6E-05	1.1E-02	-1.67	6.94E-05	5.45E-03
**DCAF15**	DDB1 and CUL4 associated factor 15	-1.07	5.0E-04	3.8E-02	-1.22	6.26E-03	4.70E-02
**DCUN1D1**	DCN1, defective in cullin neddylation 1, domain containing 1	1.13	3.3E-03	9.2E-02	1.29	3.74E-03	3.61E-02
**DGKD**	diacylglycerol kinase, delta 130kDa	-1.12	4.3E-04	3.6E-02	-1.18	1.43E-04	7.54E-03
**DHCR24**	24-dehydrocholesterol reductase	-1.19	2.3E-03	7.3E-02	-1.38	3.03E-04	1.10E-02
**DHFR**	dihydrofolate reductase	1.10	1.4E-03	5.9E-02	1.29	3.64E-04	1.20E-02
**DHRS7B**	dehydrogenase/reductase (SDR family) member 7B	-1.09	4.4E-04	3.6E-02	-1.19	1.29E-03	2.16E-02
**DNAJB5**	DnaJ (Hsp40) homolog, subfamily B, member 5	-1.14	7.0E-04	4.1E-02	-1.21	2.54E-02	9.05E-02
**DOCK1**	dedicator of cytokinesis 1	1.09	4.3E-04	3.6E-02	1.48	9.55E-05	6.37E-03
**DOCK10**	dedicator of cytokinesis 10	1.14	1.7E-03	6.4E-02	1.79	2.03E-03	2.72E-02
**DYSF**	dysferlin	-1.09	7.0E-04	4.1E-02	-1.10	2.54E-02	9.05E-02
**EBF3**	early B-cell factor 3	1.08	2.9E-04	3.1E-02	1.31	2.16E-03	2.79E-02
**ECM2**	extracellular matrix protein 2, female organ and adipocyte specific	1.14	6.9E-04	4.1E-02	1.67	1.97E-03	2.68E-02
**EDNRA**	endothelin receptor type A	1.15	3.1E-03	8.8E-02	1.61	1.80E-04	8.62E-03
**EDNRB**	endothelin receptor type B	1.19	4.3E-04	3.6E-02	1.88	1.07E-06	8.93E-04
**ELOF1**	elongation factor 1 homolog (S. cerevisiae)	-1.09	3.7E-04	3.5E-02	-1.19	1.92E-03	2.64E-02
**ENAM**	enamelin	-1.23	4.2E-04	3.6E-02	-1.20	1.67E-02	7.47E-02
**ENDOD1**	endonuclease domain containing 1	-1.12	1.2E-03	5.4E-02	-1.17	2.90E-03	3.24E-02
**ENPP2**	ectonucleotide pyrophosphatase/phosphodiesterase 2	1.27	5.0E-07	1.5E-03	1.36	8.60E-04	1.80E-02
**EPHB1**	EPH receptor B1	-1.17	9.1E-04	4.6E-02	-1.21	2.95E-02	9.71E-02
**F11R**	F11 receptor	1.11	5.2E-04	3.8E-02	1.31	2.65E-03	3.09E-02
**F2R**	coagulation factor II (thrombin) receptor	1.14	1.6E-04	2.6E-02	1.53	1.14E-05	2.59E-03
**FAM78A**	family with sequence similarity 78, member A	-1.15	3.1E-04	3.2E-02	-1.30	1.55E-03	2.36E-02
**FBXW5**	F-box and WD repeat domain containing 5	-1.10	2.4E-05	1.1E-02	-1.29	3.32E-03	3.45E-02
**FILIP1L**	filamin A interacting protein 1-like	1.11	1.2E-03	5.5E-02	1.26	2.02E-02	8.18E-02
**FLNC**	filamin C, gamma	-1.14	9.0E-04	4.6E-02	-1.20	4.70E-03	4.03E-02
**FLRT3**	fibronectin leucine rich transmembrane protein 3	-1.24	6.3E-04	4.0E-02	-1.26	1.02E-02	5.94E-02
**FRMD6**	FERM domain containing 6	1.12	1.6E-04	2.6E-02	1.51	2.15E-03	2.79E-02
**FXYD6**	FXYD domain containing ion transport regulator 6	1.08	7.4E-04	4.2E-02	1.24	1.87E-03	2.61E-02
**FZD4**	frizzled class receptor 4	1.13	1.0E-06	2.4E-03	1.35	2.13E-06	1.16E-03
**FZD6**	frizzled class receptor 6	1.13	8.8E-04	4.6E-02	1.58	1.15E-04	6.96E-03
**GABBR1**	gamma-aminobutyric acid (GABA) B receptor, 1	1.10	3.6E-03	9.5E-02	1.30	5.24E-03	4.29E-02
**GBA2**	glucosidase, beta (bile acid) 2	-1.14	5.0E-04	3.8E-02	-1.16	1.49E-02	7.07E-02
**GNAI1**	G Protein Subunit Alpha I1	1.12	1.9E-03	6.6E-02	1.51	3.22E-05	4.02E-03
**GPR133**	G protein-coupled receptor 133	-1.10	3.8E-04	3.5E-02	-1.28	2.88E-04	1.07E-02
**GRIP2**	glutamate receptor interacting protein 2	-1.12	1.7E-03	6.3E-02	-1.41	4.48E-03	3.93E-02
**GSS**	glutathione synthetase	-1.11	3.9E-05	1.4E-02	-1.24	9.63E-04	1.89E-02
**GTF2H4**	general transcription factor IIH, polypeptide 4, 52kDa	-1.07	6.2E-04	4.0E-02	-1.11	1.06E-02	6.03E-02
**GYS1**	glycogen synthase 1 (muscle)	-1.10	7.0E-04	4.1E-02	-1.17	2.70E-02	9.31E-02
**HDAC5**	histone deacetylase 5	-1.13	2.0E-05	1.1E-02	-1.24	2.69E-04	1.03E-02
**HEY1**	hes-related family bHLH transcription factor with YRPW motif 1	1.12	1.1E-03	5.3E-02	1.26	7.22E-03	5.00E-02
**HIBCH**	3-hydroxyisobutyryl-CoA hydrolase	1.13	1.3E-03	5.7E-02	1.23	5.67E-04	1.45E-02
**HIPK2**	homeodomain interacting protein kinase 2	-1.14	1.8E-04	2.6E-02	-1.32	1.05E-02	6.01E-02
**HIVEP3**	human immunodeficiency virus type I enhancer binding protein 3	-1.08	2.0E-04	2.7E-02	-1.15	1.34E-02	6.75E-02
**HOMER2**	homer homolog 2 (Drosophila)	-1.14	3.9E-04	3.5E-02	-1.28	1.52E-02	7.14E-02
**HSPB7**	heat shock 27kDa protein family, member 7 (cardiovascular)	-1.21	5.0E-05	1.6E-02	-1.29	7.10E-04	1.62E-02
**HSPG2**	heparan sulfate proteoglycan 2	1.15	4.4E-06	4.8E-03	1.38	7.78E-04	1.70E-02
**HTRA1**	HtrA serine peptidase 1	1.09	2.2E-03	7.1E-02	1.20	1.32E-02	6.71E-02
**IFI16**	interferon, gamma-inducible protein 16	1.14	3.6E-03	9.4E-02	1.45	1.61E-03	2.42E-02
**IGF1**	insulin-like growth factor 1 (somatomedin C)	1.20	5.5E-04	3.9E-02	1.71	6.44E-04	1.54E-02
**IGF2**	insulin-like growth factor 2 (somatomedin A) /// INS-IGF2 readthrough	1.27	2.5E-06	3.4E-03	1.59	2.50E-04	9.98E-03
**IGFBP5**	insulin-like growth factor binding protein 5	1.18	9.0E-04	4.6E-02	1.19	2.58E-02	9.13E-02
**IGFBP7**	insulin-like growth factor binding protein 7	1.10	3.2E-03	8.9E-02	1.41	1.83E-04	8.64E-03
**ITGB5**	integrin, beta 5	1.06	3.5E-03	9.3E-02	1.17	2.97E-03	3.28E-02
**ITPR1**	inositol 1,4,5-trisphosphate receptor, type 1	1.11	1.7E-03	6.3E-02	1.22	7.06E-03	4.97E-02
**JAM2**	junctional adhesion molecule 2	1.18	5.1E-05	1.6E-02	1.39	1.44E-05	2.72E-03
**JMJD1C**	jumonji domain containing 1C	1.09	3.0E-03	8.8E-02	1.34	4.80E-03	4.07E-02
**JPH2**	junctophilin 2	-1.11	3.4E-04	3.4E-02	-1.11	2.93E-02	9.69E-02
**JUND**	jun D proto-oncogene	-1.12	5.5E-04	3.9E-02	-1.14	2.36E-02	8.75E-02
**KCNMA1**	Potassium Calcium-Activated Channel Subfamily M Alpha 1	-1.13	3.6E-03	9.5E-02	-1.37	6.47E-04	1.54E-02
**KDR**	kinase insert domain receptor (a type III receptor tyrosine kinase)	1.16	9.7E-04	4.8E-02	1.51	2.49E-04	9.98E-03
**KLHDC2**	kelch domain containing 2	1.08	2.1E-03	7.1E-02	1.07	2.11E-02	8.31E-02
**LAMA4**	laminin, alpha 4	1.18	1.7E-04	2.6E-02	1.74	8.65E-06	2.23E-03
**LAMB1**	laminin, beta 1	1.16	4.3E-04	3.6E-02	1.68	1.61E-03	2.42E-02
**LANCL1**	LanC lantibiotic synthetase component C-like 1 (bacterial)	1.09	7.5E-04	4.3E-02	1.15	2.78E-03	3.16E-02
**LARGE**	like-glycosyltransferase	-1.09	3.2E-04	3.3E-02	-1.17	2.24E-02	8.54E-02
**LINC00312**	long intergenic non-protein coding RNA 312	-1.22	1.2E-03	5.4E-02	-1.13	2.57E-02	9.11E-02
**LOX**	lysyl oxidase	1.18	3.0E-03	8.7E-02	3.02	5.47E-05	5.00E-03
**LPIN1**	lipin 1	1.11	5.8E-04	4.0E-02	1.09	1.26E-02	6.57E-02
**LRPAP1**	low density lipoprotein receptor-related protein associated protein 1	-1.06	2.1E-03	7.0E-02	-1.17	6.41E-04	1.54E-02
**LRRFIP1**	leucine rich repeat (in FLII) interacting protein 1	-1.10	2.6E-04	3.0E-02	1.16	2.38E-02	8.79E-02
**LSM12**	LSM12 homolog (S. cerevisiae)	-1.07	1.1E-03	5.2E-02	-1.36	3.14E-04	1.11E-02
**LSMEM2**	leucine-rich single-pass membrane protein 2	-1.08	7.4E-04	4.2E-02	-1.27	2.06E-02	8.22E-02
**MAPK12**	mitogen-activated protein kinase 12	-1.10	1.2E-03	5.3E-02	-1.36	5.08E-05	4.85E-03
**MCRS1**	microspherule protein 1	-1.13	8.5E-06	7.4E-03	-1.18	1.45E-02	7.00E-02
**MCTP1**	multiple C2 domains, transmembrane 1	1.12	2.8E-03	8.4E-02	1.71	1.36E-03	2.20E-02
**MGST3**	microsomal glutathione S-transferase 3	-1.11	1.1E-03	5.1E-02	-1.06	3.14E-03	3.35E-02
**MICAL2**	microtubule associated monooxygenase, calponin and LIM domain containing 2	-1.12	8.0E-04	4.4E-02	-1.13	2.20E-02	8.48E-02
**MICAL3**	microtubule associated monooxygenase, calponin and LIM domain containing 3	-1.11	3.0E-03	8.7E-02	-1.17	2.13E-02	8.34E-02
**MMP2**	Matrix Metallopeptidase 2	1.16	2.6E-03	8.0E-02	1.27	4.35E-03	3.88E-02
**MMRN2**	multimerin 2	1.06	3.0E-03	8.7E-02	1.22	2.18E-03	2.81E-02
**MRPL23**	mitochondrial ribosomal protein L23	-1.07	2.5E-03	7.8E-02	-1.24	2.08E-03	2.75E-02
**MYADML2**	myeloid-associated differentiation marker-like 2	-1.11	8.0E-04	4.4E-02	-1.34	6.23E-04	1.52E-02
**MYL12A**	myosin, light chain 12A, regulatory, non-sarcomeric	-1.14	1.1E-03	5.2E-02	-1.07	1.78E-02	7.71E-02
**MYO1B**	myosin IB	1.22	2.3E-05	1.1E-02	1.90	1.73E-06	1.10E-03
**NAMPT**	nicotinamide phosphoribosyltransferase	1.11	1.8E-03	6.6E-02	1.26	1.39E-02	6.89E-02
**NCLN**	nicalin	-1.08	2.5E-04	3.0E-02	-1.18	1.30E-03	2.16E-02
**NETO2**	neuropilin (NRP) and tolloid (TLL)-like 2	1.11	3.9E-03	9.9E-02	1.61	6.74E-04	1.56E-02
**NFIC**	nuclear factor I/C (CCAAT-binding transcription factor)	-1.11	9.5E-05	2.0E-02	-1.26	8.89E-05	6.15E-03
**NGFRAP1**	nerve growth factor receptor (TNFRSF16) associated protein 1	1.13	3.4E-03	9.2E-02	1.26	1.65E-04	8.11E-03
**NID1**	nidogen 1	1.20	2.0E-06	3.2E-03	1.68	4.66E-06	1.76E-03
**NID2**	nidogen 2 (osteonidogen)	1.16	3.8E-04	3.5E-02	1.88	2.51E-05	3.52E-03
**NOA1**	nitric oxide associated 1	1.05	3.8E-03	9.7E-02	-1.10	5.09E-03	4.22E-02
**NOTCH4**	notch 4	1.08	2.9E-03	8.5E-02	1.48	1.31E-05	2.71E-03
**NQO1**	NAD(P)H dehydrogenase, quinone 1	1.20	8.9E-04	4.6E-02	1.34	4.62E-04	1.32E-02
**NQO2**	NAD(P)H dehydrogenase, quinone 2	-1.08	6.3E-04	4.0E-02	-1.24	6.39E-04	1.54E-02
**NR1H2**	nuclear receptor subfamily 1, group H, member 2	-1.08	2.1E-03	7.0E-02	-1.27	1.74E-03	2.51E-02
**NRAP**	nebulin-related anchoring protein	-1.07	1.1E-03	5.1E-02	-1.16	2.43E-04	9.93E-03
**NTN4**	netrin 4	1.08	1.6E-03	6.3E-02	1.20	1.77E-02	7.68E-02
**NUTF2**	nuclear transport factor 2	1.12	3.4E-03	9.2E-02	-1.12	2.46E-02	8.92E-02
**OLFML2B**	olfactomedin-like 2B	1.25	4.5E-06	4.8E-03	1.45	1.45E-03	2.28E-02
**OR2A20P**	olfactory receptor, family 2, subfamily A, member 20 pseudogene	1.14	1.8E-04	2.6E-02	1.37	3.26E-04	1.15E-02
**OTUB1**	OTU Deubiquitinase, Ubiquitin Aldehyde Binding 1	-1.07	1.8E-03	6.4E-02	-1.21	3.04E-03	3.30E-02
**P4HB**	prolyl 4-hydroxylase, beta polypeptide	-1.05	3.0E-03	8.7E-02	-1.11	4.13E-03	3.78E-02
**PAG1**	phosphoprotein membrane anchor with glycosphingolipid microdomains 1	1.10	5.7E-04	4.0E-02	1.57	5.66E-06	1.80E-03
**PARP4**	poly (ADP-ribose) polymerase family, member 4	1.11	6.5E-04	4.1E-02	1.20	1.65E-02	7.45E-02
**PCBP4**	poly(rC) binding protein 4	-1.10	7.9E-04	4.4E-02	-1.28	1.28E-03	2.15E-02
**PCMTD1**	protein-L-isoaspartate (D-aspartate) O-methyltransferase domain containing 1	1.14	1.5E-04	2.6E-02	1.15	2.08E-03	2.75E-02
**PCNT**	pericentrin	-1.11	3.3E-04	3.3E-02	-1.12	4.24E-03	3.83E-02
**PDGFRB**	platelet-derived growth factor receptor, beta polypeptide	1.11	1.8E-03	6.5E-02	1.37	1.36E-05	2.71E-03
**PECAM1**	platelet/endothelial cell adhesion molecule 1	1.20	1.3E-04	2.4E-02	1.40	1.17E-03	2.06E-02
**PGP**	phosphoglycolate phosphatase	-1.13	2.6E-04	3.0E-02	-1.22	2.46E-03	2.97E-02
**PKI55**	DKFZp434H1419	-1.11	4.0E-04	3.5E-02	-1.16	2.70E-02	9.32E-02
**PLXNA1**	plexin A1	-1.09	2.3E-03	7.4E-02	-1.12	1.29E-02	6.63E-02
**PON2**	paraoxonase 2	1.10	3.5E-03	9.3E-02	1.29	3.89E-04	1.24E-02
**PPP1R15A**	protein phosphatase 1, regulatory subunit 15A	-1.16	7.6E-05	1.8E-02	-1.26	2.57E-02	9.11E-02
**PPP2R3B**	Protein Phosphatase 2 Regulatory Subunit B''Beta	-1.07	9.8E-04	4.8E-02	-1.17	8.93E-03	5.59E-02
**PRCP**	prolylcarboxypeptidase (angiotensinase C)	1.17	3.3E-04	3.3E-02	1.31	2.16E-04	9.44E-03
**PREP**	prolyl endopeptidase	1.07	9.2E-04	4.6E-02	1.16	1.73E-02	7.61E-02
**PROX1**	prospero homeobox 1	-1.18	5.9E-04	4.0E-02	-1.55	1.40E-02	6.90E-02
**PTGR1**	prostaglandin reductase 1	1.12	3.2E-03	9.1E-02	1.18	1.27E-02	6.58E-02
**PTPN14**	protein tyrosine phosphatase, non-receptor type 14	-1.10	5.1E-04	3.8E-02	-1.24	6.00E-03	4.59E-02
**PXDN**	peroxidasin homolog (Drosophila)	1.17	1.7E-04	2.6E-02	1.94	4.71E-06	1.76E-03
**QKI**	QKI, KH domain containing, RNA binding	-1.10	5.8E-05	1.6E-02	1.14	5.26E-03	4.30E-02
**RAB30**	RAB30, member RAS oncogene family	1.15	4.8E-04	3.7E-02	1.16	2.70E-02	9.31E-02
**RAB31**	RAB31, member RAS oncogene family	1.13	1.4E-03	5.9E-02	1.26	8.30E-03	5.37E-02
**RBM27**	RNA binding motif protein 27	-1.07	3.1E-03	8.8E-02	-1.11	2.94E-02	9.71E-02
**RBP1**	retinol binding protein 1, cellular	1.14	3.5E-03	9.3E-02	1.51	2.05E-04	9.20E-03
**RBP7**	retinol binding protein 7, cellular	1.13	3.8E-03	9.7E-02	1.53	1.29E-04	7.36E-03
**RCBTB2**	RCC1 And BTB Domain Containing Protein 2	1.13	9.0E-04	4.6E-02	1.22	7.52E-03	5.09E-02
**RECK**	reversion-inducing-cysteine-rich protein with kazal motifs	1.10	2.0E-03	6.8E-02	1.23	1.30E-03	2.16E-02
**RFTN2**	raftlin family member 2	1.20	1.6E-05	1.1E-02	1.74	9.55E-05	6.37E-03
**RHOJ**	ras homolog family member J	1.13	2.7E-04	3.0E-02	1.31	2.14E-04	9.41E-03
**RNF122**	ring finger protein 122	-1.21	1.0E-03	4.9E-02	-1.20	5.20E-03	4.26E-02
**RPL18A**	ribosomal protein L18a /// small nucleolar RNA, H/ACA box 68	-1.05	1.7E-03	6.3E-02	-1.11	2.36E-04	9.80E-03
**RPLP0**	ribosomal protein, large, P0	-1.08	2.6E-04	3.0E-02	-1.04	9.91E-04	1.91E-02
**RQCD1**	RCD1 required for cell differentiation1 homolog (S. pombe)	-1.10	3.1E-04	3.2E-02	-1.21	2.82E-02	9.52E-02
**SASH1**	SAM and SH3 domain containing 1	1.14	4.5E-04	3.6E-02	1.46	2.59E-05	3.57E-03
**SCAP**	SREBF chaperone	-1.06	1.9E-03	6.7E-02	-1.20	4.30E-04	1.28E-02
**SCN7A**	sodium channel, voltage-gated, type VII, alpha subunit	1.20	6.8E-04	4.1E-02	1.30	1.44E-03	2.28E-02
**SEMA5A**	Semaphorin 5A	1.10	5.3E-04	3.9E-02	1.32	6.45E-03	4.77E-02
**SEMA6A**	Semaphorin 6A	1.14	7.9E-04	4.4E-02	1.29	1.44E-04	7.54E-03
**SETD7**	SET domain containing (lysine methyltransferase) 7	1.11	6.0E-05	1.6E-02	1.16	6.27E-03	4.70E-02
**SH3BP5**	SH3-domain binding protein 5 (BTK-associated)	1.09	5.9E-04	4.0E-02	1.21	7.11E-03	4.97E-02
**SIPA1L2**	signal-induced proliferation-associated 1 like 2	1.15	9.6E-04	4.8E-02	1.46	1.26E-03	2.13E-02
**SLC1A7**	solute carrier family 1 (glutamate transporter), member 7	1.11	1.8E-04	2.6E-02	1.26	1.39E-02	6.89E-02
**SLC25A37**	solute carrier family 25 (mitochondrial iron transporter), member 37	-1.09	2.1E-04	2.7E-02	-1.09	2.01E-02	8.17E-02
**SLC9A5**	Solute Carrier Family 9 Member A5	-1.09	3.8E-04	3.5E-02	-1.24	3.58E-03	3.56E-02
**SLFN5**	schlafen family member 5	1.09	1.5E-03	5.9E-02	1.21	7.73E-04	1.70E-02
**SLN**	sarcolipin	1.07	1.4E-04	2.5E-02	1.04	6.68E-03	4.84E-02
**SMOC2**	SPARC related modular calcium binding 2	1.18	6.0E-04	4.0E-02	1.27	1.17E-03	2.06E-02
**SNAI2**	snail family zinc finger 2	1.15	1.9E-04	2.6E-02	1.54	5.16E-05	4.86E-03
**SNTA1**	syntrophin, alpha 1	-1.11	1.1E-03	5.2E-02	-1.30	4.24E-04	1.27E-02
**SNTB1**	Syntrophin Beta 1	1.11	1.9E-03	6.7E-02	1.24	1.47E-02	7.06E-02
**SPARC**	secreted protein, acidic, cysteine-rich (osteonectin)	1.33	1.9E-06	3.2E-03	1.66	2.35E-08	1.77E-04
**SPARCL1**	SPARC-like 1 (hevin)	1.17	1.1E-04	2.2E-02	1.29	1.66E-04	8.15E-03
**SPIN1**	spindlin 1	1.13	6.6E-05	1.6E-02	1.34	1.81E-04	8.63E-03
**SRGAP3**	SLIT-ROBO Rho GTPase activating protein 3	-1.08	8.4E-04	4.5E-02	-1.30	1.26E-03	2.13E-02
**STARD9**	StAR-related lipid transfer (START) domain containing 9	1.11	3.1E-03	8.8E-02	1.36	4.75E-05	4.74E-03
**STOM**	stomatin	1.20	8.0E-05	1.8E-02	1.19	1.17E-03	2.06E-02
**STX2**	syntaxin 2	1.12	8.0E-04	4.4E-02	1.22	2.12E-03	2.77E-02
**SULF2**	sulfatase 2	1.10	4.6E-04	3.6E-02	1.31	6.89E-05	5.45E-03
**SURF1**	surfeit 1	-1.09	1.7E-03	6.4E-02	-1.13	4.54E-04	1.30E-02
**SVEP1**	sushi, von Willebrand factor type A, EGF and pentraxin domain containing 1	1.16	3.5E-03	9.3E-02	1.22	1.82E-02	7.79E-02
**TBL3**	transducin (beta)-like 3	-1.06	3.9E-03	9.9E-02	-1.15	3.54E-03	3.54E-02
**TFRC**	transferrin receptor	-1.28	4.6E-04	3.6E-02	-1.40	4.51E-03	3.94E-02
**THOP1**	thimet oligopeptidase 1	-1.07	1.3E-03	5.7E-02	-1.18	9.40E-04	1.87E-02
**THY1**	Thy-1 cell surface antigen	1.17	2.0E-03	6.8E-02	2.06	1.41E-05	2.71E-03
**TMEM120B**	transmembrane protein 120B	-1.09	1.8E-03	6.5E-02	-1.17	1.37E-02	6.83E-02
**TMEM243**	transmembrane protein 243, mitochondrial	1.09	2.4E-03	7.6E-02	1.20	1.12E-02	6.20E-02
**TMTC1**	transmembrane and tetratricopeptide repeat containing 1	-1.12	5.2E-04	3.8E-02	-1.17	9.33E-03	5.70E-02
**TNFSF10**	tumor necrosis factor (ligand) superfamily, member 10	1.16	2.4E-03	7.7E-02	1.27	9.86E-03	5.84E-02
**TNRC18**	trinucleotide repeat containing 18	-1.10	1.3E-03	5.7E-02	-1.15	2.01E-02	8.17E-02
**TRPT1**	tRNA phosphotransferase 1	1.09	1.4E-03	5.7E-02	1.18	1.61E-02	7.37E-02
**TUBA4A**	tubulin, alpha 4a	-1.30	5.0E-08	3.7E-04	-1.23	9.19E-06	2.29E-03
**TUBA4B**	tubulin, alpha 4b (pseudogene)	-1.11	2.0E-04	2.6E-02	-1.34	6.02E-04	1.50E-02
**TUBB**	tubulin, beta class I	-1.15	1.8E-04	2.6E-02	-1.17	2.66E-04	1.03E-02
**TUBB4B**	tubulin, beta 4B class IVb	-1.19	1.2E-05	8.6E-03	-1.22	2.34E-04	9.77E-03
**TUBG1**	tubulin, gamma 1	-1.14	2.6E-04	3.0E-02	-1.24	2.07E-03	2.74E-02
**TUSC3**	tumor suppressor candidate 3	1.11	3.5E-03	9.3E-02	1.59	1.59E-05	2.80E-03
**TXLNG**	taxilin gamma	-1.07	1.7E-03	6.3E-02	1.19	1.69E-02	7.53E-02
**UACA**	uveal autoantigen with coiled-coil domains and ankyrin repeats	1.16	4.1E-06	4.8E-03	1.44	9.28E-03	5.69E-02
**UBE2Q2**	ubiquitin-conjugating enzyme E2Q family member 2	1.15	9.0E-04	4.6E-02	1.16	1.31E-02	6.66E-02
**UNK**	unkempt family zinc finger	-1.08	3.8E-04	3.5E-02	-1.15	2.44E-02	8.89E-02
**USP2**	ubiquitin specific peptidase 2	-1.16	8.1E-05	1.8E-02	-1.20	1.66E-02	7.45E-02
**USP54**	ubiquitin specific peptidase 54	-1.23	2.4E-05	1.1E-02	-1.23	2.65E-02	9.23E-02
**UTRN**	utrophin	1.10	3.5E-03	9.3E-02	1.42	3.87E-04	1.24E-02
**VIPR2**	vasoactive intestinal peptide receptor 2	-1.18	7.3E-05	1.8E-02	-1.34	5.72E-04	1.46E-02
**VPS8**	vacuolar protein sorting 8 homolog (S. cerevisiae)	-1.12	3.3E-03	9.2E-02	-1.24	6.58E-03	4.81E-02
**WBP2**	WW domain binding protein 2	-1.09	2.4E-06	3.4E-03	-1.20	1.33E-03	2.18E-02
**WDR1**	WD repeat domain 1	-1.10	1.7E-03	6.4E-02	-1.23	1.40E-04	7.51E-03
**XAF1**	XIAP associated factor 1	1.18	2.7E-03	8.2E-02	1.80	9.01E-04	1.83E-02
**XIRP1**	xin actin-binding repeat containing 1	-1.43	5.3E-05	1.6E-02	-1.25	1.64E-02	7.43E-02
**ZDHHC3**	zinc finger, DHHC-type containing 3	-1.09	1.3E-03	5.7E-02	-1.22	2.85E-04	1.06E-02
**ZNF521**	zinc finger protein 521	1.16	3.1E-04	3.2E-02	1.33	9.75E-03	5.80E-02
**ZNF83**	zinc finger protein 83	1.16	3.9E-04	3.5E-02	1.50	2.54E-02	9.06E-02
